# Supplementary material for: Acute Cardiac Tamponade: An Adult Simulation Case for Residents
Source: MedEdPORTAL. 2016 Sep 23;12:10466. doi: 10.15766/mep_2374-8265.10466 (PMC6464418; doi:10.15766/mep_2374-8265.10466)
Supplement: Supplementary file 1 — A. Simulation Case.docx B. PowerPoint Presentation.pptx C. Critical Actions Checklist.docx D. Assessment of a Low-Cost Ultrasound Pericardiocentesis Model.pdf [file mep-12-10466-s001.zip › _Educational Summary Report_10466.pdf]

MedEdPORTAL®
The Journal of Teaching and Learning Resources

Crossref

Original Publication

OPEN ACCESS

Acute Cardiac Tamponade: An Adult Simulation Case for Residents

Mohammed Alkhalifah, MD*, Michelle McLean, MD, Ahmad Koshak, MD
*Corresponding author: alkhalifaa2@gmail.com

Citation: Alkhalifah M, McLean M, Koshak A. Acute cardiac tamponade: an adult simulation case for residents. *MedEdPORTAL*. 2016;12:10466. https://doi.org/10.15766/mep_2374-8265.10466

Copyright: © 2016 Alkhalifah et al. This is an open-access article distributed under the terms of the Creative Commons Attribution license.

Appendices

A. Simulation Case.docx
B. PowerPoint Presentation .pptx
C. Critical Actions Checklist .docx
D. Assessment of a Low-Cost Ultrasound Pericardiocentesis Model .pdf

All appendices are peer reviewed as integral parts of the Original Publication.

Abstract

Introduction: This case was designed to be an interprofessional scenario to assist the learner in the approach to a patient with cardiac tamponade. This resource provides information and materials for a high-fidelity simulation scenario that is appropriate for learners at multiple levels. Each learner has the opportunity to interact with embedded patient actors. This scenario includes the opportunity to conduct a bedside echocardiogram and perform an ultrasound-guided pericardiocentesis. **Methods:** A 51-year-old female presents to the emergency department with a chief complaint of shortness of breath and with a history of cancer. Her shortness of breath evolves into tachypnea, hypoxia, altered mental status, hypotension, and shock. The physical assessment reveals hypotension, muffled heart tones, and jugular vein distension. One critical action requires the recognition of pericardial effusion on a bedside echocardiogram. The learner needs to interpret the ultrasound findings in conjunction with the physical exam findings to diagnose cardiac tamponade. An emergent ultrasound-guided pericardiocentesis is to be performed. Equipment needed includes a high-fidelity adult mannequin, the low-cost ultrasound pericardiocentesis model (or pericardocentesis model of choice), bedside ultrasound, the PowerPoint presentation containing imaging, and laboratory data. **Results:** A survey was completed by 23 residents who participated in this simulation. Ninety-five percent of those responding felt that this simulation in conjunction with the debriefing was effective in managing a patient presenting with undifferentiated shortness of breath and pericardial tamponade. **Discussion:** This case will be incorporated into the 3-year curriculum for our emergency medicine residency program and assist with evaluation of resident performance of pericardiocentesis.

Keywords

Ultrasound, Pericardiocentesis, Emergency Medicine, Critical Care, Task Trainer, Cardiac Tamponade, High-Fidelity Simulation, Echo

Educational Objectives

By the end of the simulation, the learner will be able to:

1. Demonstrate the appropriate initial approach to a patient presenting with difficulty breathing.
2. Construct a differential diagnosis that includes cardiac tamponade.
3. List appropriate tests for cardiac tamponade.
4. Categorize the patient as hemodynamically unstable.
5. Justify/conclude the need for pericardiocentesis.
6. Demonstrate closed-loop communication for orders.
7. Perform a complete physical exam.
8. Interpret a bedside/portable echocardiogram.

Introduction

Prognosis of cardiac tamponade depends on early recognition and treatment. This condition can be rapidly fatal if it is not considered early in the patient encounter. Learners may not encounter a case of pericardial tamponade in clinical practice during their training.[1-3] In addition, pericardiocentesis is a low-occurrence, high-risk procedure. It is performed infrequently, and therefore, providers may lose proficiency. This case fits perfectly into simulation training. We searched the MedEdPORTAL Publications database and found no case that fits this description. This prompted the development of a high-fidelity simulation case based on a real patient encounter. This resource provides the key materials for a high-fidelity simulation case that is appropriate for emergency medicine rotating medical students and residents, critical care providers and fellows, nurse practitioners, and physician assistants practicing in the emergency department environment. Providers should have a solid background in history and physical exam skills. Learners should have a basic understanding of the pathophysiology of the respiratory and cardiac systems. They should be familiar with the interpretation of echocardiograms, chest imaging, and laboratory values. This case allows learners the opportunity to utilize bedside ultrasound to perform ultrasound-guided pericardiocentesis, which is the current recommended practice. This case requires the learner to utilize critical thinking skills and demands integration of the history, physical assessment, and adjunct tests to effectively arrive at the diagnosis and provide emergent treatment.

Methods

This simulation case consists of five files that can be implemented by faculty in either a simulation lab or an in situ environment. The simulation case file (Appendix A) includes all the information to plan and implement the case. A high-fidelity mannequin/simulator should be used for the patient. Any pericardoicentesis model may be used for this case. An article describing the construction of a low-cost model is included with this case (Appendix D).[4] The Dell'Orto et al. model comes with full instructions and is economical as compared to commercial models (e.g., the Blue Phantom pericardiocentesis model costs $17,160).[5] The model referenced in our case costs approximately $68 and can be assembled using readily available materials in approximately 120 minutes. This model was selected for the case as the budget did not allow for purchase of a commercial model. Please be aware that commercial models and other articles discussing low-cost models are also available.[5,6] The pericardiocentesis task trainer is covered by a sheet in the simulation room. The embedded nurse actor facilitating the case uncovers the trainer once the learner makes the decision to perform a bedside pericardiocentesis.

This case can be presented with two faculty members. One faculty member may be the voice of the patient and answer questions relating to the history and physical assessment. The second faculty member may be the nurse in the room who provides the test results. Description for additional actors is included in the simulation case.

A PowerPoint presentation (Appendix B) is included for use during the simulation. It provides information that would be present on a triage-nursing note. It also contains the pertinent adjuncts to this case, including echocardiogram, chest radiograph, bedside ultrasound (video and still image), and laboratory findings. A critical actions checklist (Appendix C) is also included. This scenario is designed to be used as a formative teaching tool. The critical actions may be used to guide the debriefing period. This critical action checklist is not designed to be a summative evaluation tool.

Results

A survey was completed by 23 residents who participated in this simulation during the emergency medicine skills day. Ninety-five percent of those responding felt that this simulation in conjunction with the debriefing was effective in managing a patient presenting with undifferentiated shortness of breath and pericardial tamponade.

There were five groups that completed this scenario on the same day. Our experience to date has been that the learner may not be able to diagnose pericardial tamponade based on clinical evaluation only. Three groups failed to diagnose pericardial tamponade. During the debriefing session, it was apparent that they did not realize the significance of the patient's cancer history with chemotherapy and radiation. These three groups also did not utilize bedside ultrasound in this undifferentiated patient. The two groups that made the decision to utilize bedside ultrasound early in the course of their evaluation were able to diagnose and effectively treat the cardiac tamponade.

At the end of the scenario, all five groups of learners recognized the utility of bedside ultrasound early during the evaluation of a patient with undifferentiated shortness of breath. Providers must be vigilant in taking a complete history, performing a thorough physical assessment, and utilizing bedside ultrasound to assist them with the prompt diagnosis of critical medical conditions.

Discussion

This scenario details a patient who presents with a history and physical examination consistent with pericardial tamponade. The simulation allows for providers to manage a case that is low occurrence yet high risk. The strengths of the case include the ability to utilize bedside ultrasound to aid in the diagnosis and the opportunity to perform an ultrasound-guided pericardiocentesis.

A limitation of this high-fidelity simulation case is that it is not possible to fully encompass each of the specific causes of pericardial tamponade during one training session. Another limitation is that the high-fidelity simulator does not demonstrate the physical fidelity of jugular vein distention or muffled heart tones. Staff must verbalize these as the learner evaluates the neck and listens to heart tones. In addition, learners felt the amount of time to perform the pericardiocentesis was not adequate for mastery of this procedure. A task-training station for pericardiocentesis will be added during future skills sessions.

Mohammed Alkhalifah, MD: Neurocritical Care Fellow, University of Chicago Division of the Biological Sciences The Pritzker School of Medicine

Michelle McLean, MD: Assistant Director of the Clinical Simulation Center, Central Michigan University College of Medicine

Ahmad Koshak, MD: Assistant Professor of Emergency Medicine, Central Michigan University College of Medicine

---

Disclosures
None to report.

Funding/Support
None to report.

Ethical Approval
Reported as not applicable.

References

1. Palmer JD. Chest pain. In: Sherman SC, Weber JM, Schindlbeck MA, Patwari RG, eds. *Clinical Emergency Medicine*. New York, NY: McGraw-Hill; 2014:46-49.
2. Shiber JR. Pericardial diseases. In: Farcy DA, Chiu WC, Flaxman A, Marshall JP, eds. *Critical Care Emergency Medicine*. New York, NY: McGraw-Hill; 2012:181-192.
3. Niemann JT. Cardiomyopathies and pericardial disease. In: Tintinalli JE, Stapczynski JS, Ma OJ, Yealy DM, Meckler GD, Cline DM, eds. *Tintinalli's Emergency Medicine: A Comprehensive Study Guide*. 8th ed. New York, NY: McGraw-Hill; 2016:380-387.
4. Saito Y, Donohue A, Attai S, et al. The syndrome of cardiac tamponade with "small" pericardial effusion. *Echocardiography*. 2008;25(3):321-327. http://dx.doi.org/10.1111/j.1540-8175.2007.00567.x

5. Transthoracic echocardiography and pericardiocentesis ultrasound training model. CAE Healthcare Web site. http://www.bluephantom.com/product/Transthoracic-Echocardiography-and-Pericardiocentesis-Ultrasound-Training-Model.aspx?cid=411
6. Daly RJ, Planas JH, Edens MA. Adapting gel-wax into an ultrasound guided pericardiocentesis model at low cost. Poster presented at: Council of Emergency Medicine Residency Directors Academic Assembly; March 6-9, 2016; Nashville, TN. http://www.cordem.org/i4a/pages/index.cfm?pageid=3996

Received: February 9, 2015 | **Accepted:** August 12, 2016 | **Published:** September 23, 2016
