# Supplementary material for: Acute Cardiac Tamponade: An Adult Simulation Case for Residents
Source: MedEdPORTAL. 2016 Sep 23;12:10466. doi: 10.15766/mep_2374-8265.10466 (PMC6464418; doi:10.15766/mep_2374-8265.10466)
Supplement: Supplementary file 1 — A. Simulation Case.docx B. PowerPoint Presentation.pptx C. Critical Actions Checklist.docx D. Assessment of a Low-Cost Ultrasound Pericardiocentesis Model.pdf [file mep-12-10466-s001.zip › A. Simulation Case.docx]

| **Appendix A: MedEdPORTAL Simulation Case Template**  **SIMULATION CASE TITLE:** " Acute Cardiac Tamponade: An Adult  Simulation Case for Residents"  **AUTHORS: Mohammed Alkhalifah, MBBS, Ahmad Koshak, MD, Michelle McLean, MD, EMT-P FACEP** | |
| --- | --- |
| **PATIENT NAME: Julia**  **PATIENT AGE: 51 year old female**  **CHIEF COMPLAINT: Shortness of breath** | |
|  | |
| **Brief narrative description of case** | Pt arrived via triage for cough, difficulty breathing and wheezing. Pt states she has not been able to eat lately either. |
| **Primary Learning Objectives** | 1. Primary : 2. Demonstrate the appropriate initial approach to a patient presenting with difficulty breathing. (Application) 3. Construct a differential diagnosis that includes cardiac tamponade. (Application) 4. List appropriate tests for cardiac tamponade. (Remembering) 5. Categorizes the patient as hemodynamically unstable. (Analyzing) 6. Justifies/concludes the need for pericardiocentesis. (Evaluating) 7. **Secondary:** 8. Demonstrate closed loop communication for orders. (**Application**) 9. Perform a complete physical exam. (**Application**) 10. Interpret a bedside/portable echocardiogram. (**Analyzing**) |
| **Critical Actions** | 1. Evaluate airway, breathing and circulation. 2. Apply patient to cardiac monitor, pulse oximeter. 3. Establish peripheral access. 4. Apply supplemental oxygen. 5. Obtain Past Medical History. 6. Obtain complete physical examination. 7. Analyze the significance of pertinent physical examination findings including: tachycardia, hypotension, distant heart sounds, JVD. 8. Order portable chest radiograph. 9. Recognize significant cardiomegaly on chest radiograph. 10. Order an EKG. 11. Recognize and discuss electrical alternans. 12. Perform bedside ECHO. 13. Interpret ECHO, recognize pericardial effusion. 14. Performing emergent pericardiocentesis. (Learner should consult specialist if unfamiliar with the procedure.) 15. Admit patient to ICU, contact appropriate consultants, cardiology, and cardiothoracic surgery. |
| **Learner Preparation** | - Tour of sim lab. - Introduction about the high fidelity simulation for learners. - Triage report with vital signs. |

| Initial Presentation | | | |
| --- | --- | --- | --- |
| **Initial vital signs** | VS: Temp. 37.2 ◦C (99 ◦F), HR 124, RR 22, BP 117/74, O2 sat 96% RA. | | |
| **Overall Appearance** | Middle aged female, anxious, diaphoretic, in moderate respiratory distress. | | |
| **Actors and roles in the room at case start** | - Patient voice is female and should demonstrate shortness of breath. (Incomplete sentences.) - A family member: anxious, keeps questioning the learner about the treatment plan. Once reassured remains calm. (Action role: Arriving with patient “This is her asthma acting up. All she needs is a breathing treatment” They should question the diagnosis of tamponade and the urgency to do pericardiocentesis. “Are you putting a hole in her heart??) - ED nurse can start IV and assist as requested. - Cardiologist:  available by phone for consultation. | | |
| HPI | This is a 51-year-old female presenting to the emergency department with a chief complaint of shortness of breath. The patient has past medical history significant for cancer, status post chemo and radiation therapy. The patient has past medical history significant for asthma and diabetes. She was in her usual state of health until 2 weeks ago when she started to complain of worsening shortness of breath. This is exertional, associated with wheezing and a productive cough. The patient denies hemoptysis. The patient denies chest pain. She complains of chest tightness. She is also complaining of abdominal pain located in the right upper quadrant. This pain is chronic in nature and does not radiate. It is not associated with diarrhea or vomiting. The patient has used albuterol treatments over the last 2 weeks with minimal relief. Patient denies recent travel or hospitalization. She denies history of blood clots or pulmonary embolism. She states this is very different than her previous asthma exacerbations. | | |
| **Past Medical/Surgical History** | **Medications** | **Allergies** | **Family/social History** |
| - Diabetes mellitus - Bronchial asthma - Depression - Ovarian cancer s/p chemo and radiation therapy last dose one year ago. | - Aspirin - Hydrocodone - Insulin - Zofran | - No known allergies | Never smoked tobacco, no drug abuse |
| **Physical Examination** | | | |
| **General** | Middle aged female, anxious, diaphoretic, in moderate respiratory distress. | | |
| **HEENT** | Normocephalic and atraumatic, conjunctivae and EOM are normal. | | |
| **Neck** | Normal range of motion. Negative for stridor, JVD (a clinical picture for JVD is included in appendix B) | | |
| **Lungs** | Air entry is diminished bilaterally. There is end expiratory wheezing in all lung fields. | | |
| **Cardiovascular** | Regular rhythm and intact distal pulses. Tachycardia is present. Exam reveals distant heart sounds/muffled heart tones. (This may be verbalized by the embedded actor representing the nurse as the learner auscultates heart tones.) | | |
| **Abdomen** | Soft. Bowel sounds are normal. There is non-localized abdominal tenderness. There are no peritoneal signs. | | |
| **Neurological** | She is alert and oriented to person, place, and time. She has normal strength. No cranial nerve deficit or sensory deficit. | | |
| **Skin** | No rash noted. | | |
| **GU** | No rash, no discharge. | | |
| **Psychiatric** | Anxious. | | |

| Instructor Notes - Changes and CASE Branch Points*.* | | |
| --- | --- | --- |
| **Intervention / Time point** | **Change in Case** | **Additional Information** |
| Initial assessment | No change in vital signs. | Allow learner 5 minutes to obtain focused history and examination. |
| 5 minutes | Decrease in oxygenation to 88% if no oxygen supplementation is provided. Hypotension will ensue, BP 90/50, if no IV fluids given. Wheezing resolves if bronchodilator given but shortness of breath continues | Vital signs will remain unchanged with supplemental oxygen and fluids administration. Wheezing resolves with bronchodilator therapy. Shortness of breath continues despite these therapies. |
| 10 minutes | Initial work up preformed (12 leads EKG, portable CXR, bedside echo). | Learner should recognize pericardial tamponade. The embedded actor (RN) may prompt clinical findings if the learner struggles. |
| 11 minutes | Pt will start to become tachypneic, hypotensive, hypoxic with altered mental status. | The learner should verbalize the need for emergent pericardiocentesis. (The procedure should be performed or verbalized if the task trainer is not available.) |
| 11 minutes | If the tamponade was not recognized the patient will develop PEA arrest.  (The embedded nurse actor may assist learner by prompting the past medical history of cancer, and the abnormal physical exam findings. Muffled heart tones, JVD, dyspnea, hypotension.) | If PEA arrest occurs, allow ACLS resuscitation for 2 minutes then terminate case for debriefing. |
| 12 minutes | If learner wants to perform procedure the family member will question the procedure and their abilities. | A family member: anxious, keeps questioning the learner about the treatment plan. Once reassured remains calm. (Action role: Arriving with patient “this is her asthma acting up. All she needs is a breathing treatment.” They will question the diagnosis of tamponade and the urgency to do pericardiocentesis. “What does that mean?”, “Are you putting a hole in her heart??”) |
| 13 minutes | Patient vital signs will stabilize after performing the procedure (returning to initial presenting setting). |  |
| Calling for disposition and consultation | Final disposition should be admission in CCU with cardiac and/or cardiothoracic consultation. |  |

**Ideal Scenario Flow**

The patient is complaining of SOB. She will have sinus tachycardia on the cardiac monitor. Tachycardia will not respond to volume resuscitation (HR 115-130). Wheezing will improve with bronchodilators (if given). Shortness of breath will persist. If the patient did not receive oxygen support and fluid resuscitation, the patient will start to deteriorate and become hypoxic and less responsive. A STAT 12 lead EKG should be obtained and reviewed. EKG will show electrical alternans (The embedded nurse actor may prompt the learner to this finding if it is missed.) Learners should verbalize the diagnosis of pericardial tamponade and/or the need for advanced imaging. The patient will start to become tachypneic, hypotensive, hypoxic and altered. A bedside ultrasound should be performed. The pericardiocentesis trainer will be present and covered in the simulation room. The embedded nurse actor facilitating the case will uncover the trainer once the learner makes the decision to perform a bedside pericardiocentesis. The learner should verbalize the need for emergent pericardiocentesis. The procedure should be performed or verbalized if the task trainer is not available.   If the tamponade was not recognized the patient will develop PEA arrest (the embedded nurse actor may prompt the learner to the EKG, Vital signs, history of cancer, and the physical exam findings to assist with the diagnosis). Final disposition should be admission in CCU with cardiac consultation.

**Anticipated Management Mistakes**

- Instructors can directly influence the flow of the scenario by providing the initial patient history via both nursing and paramedics report.  They may reintroduce these facts if the learner struggles with making the correct diagnosis.
- The physical exam findings will be verbalized when the learner verbalizes looking at the neck and as they auscultate heart tones. The embedded actor may prompt these findings if the learner struggles to make the correct diagnosis.
- Failure to recognize tamponade physiology: We recognize that this case can be challenging.  If the learner struggles with getting the diagnosis the embedded actor may prompt the learner to the history of cancer and the pertinent physical exam findings. If these prompts are required this may be addressed in the debrief session. The combination of clinical findings (low arterial blood pressure, distended neck veins and distant, muffled heart sounds), EKG, CXR and bedside 2D echo will direct the learner to the correct diagnosis.
- Learners may not be familiar with bedside ultrasound. If this is the case you can provide the video and help them with interpretations (they can ask for an echocardiography technician or cardiology consultation).
- Time will be allowed at the end of simulation conference to practice the procedure and ultrasound. This can be supervised by faculty or senior residents/fellows.
- Discuss the scenario with actors prior to simulation sessions.
