# Supplementary material for: Acute Cardiac Tamponade: An Adult Simulation Case for Residents
Source: MedEdPORTAL. 2016 Sep 23;12:10466. doi: 10.15766/mep_2374-8265.10466 (PMC6464418; doi:10.15766/mep_2374-8265.10466)
Supplement: Supplementary file 1 — A. Simulation Case.docx B. PowerPoint Presentation.pptx C. Critical Actions Checklist.docx D. Assessment of a Low-Cost Ultrasound Pericardiocentesis Model.pdf [file mep-12-10466-s001.zip › B. PowerPoint Presentation.pptx]

## Slide 1
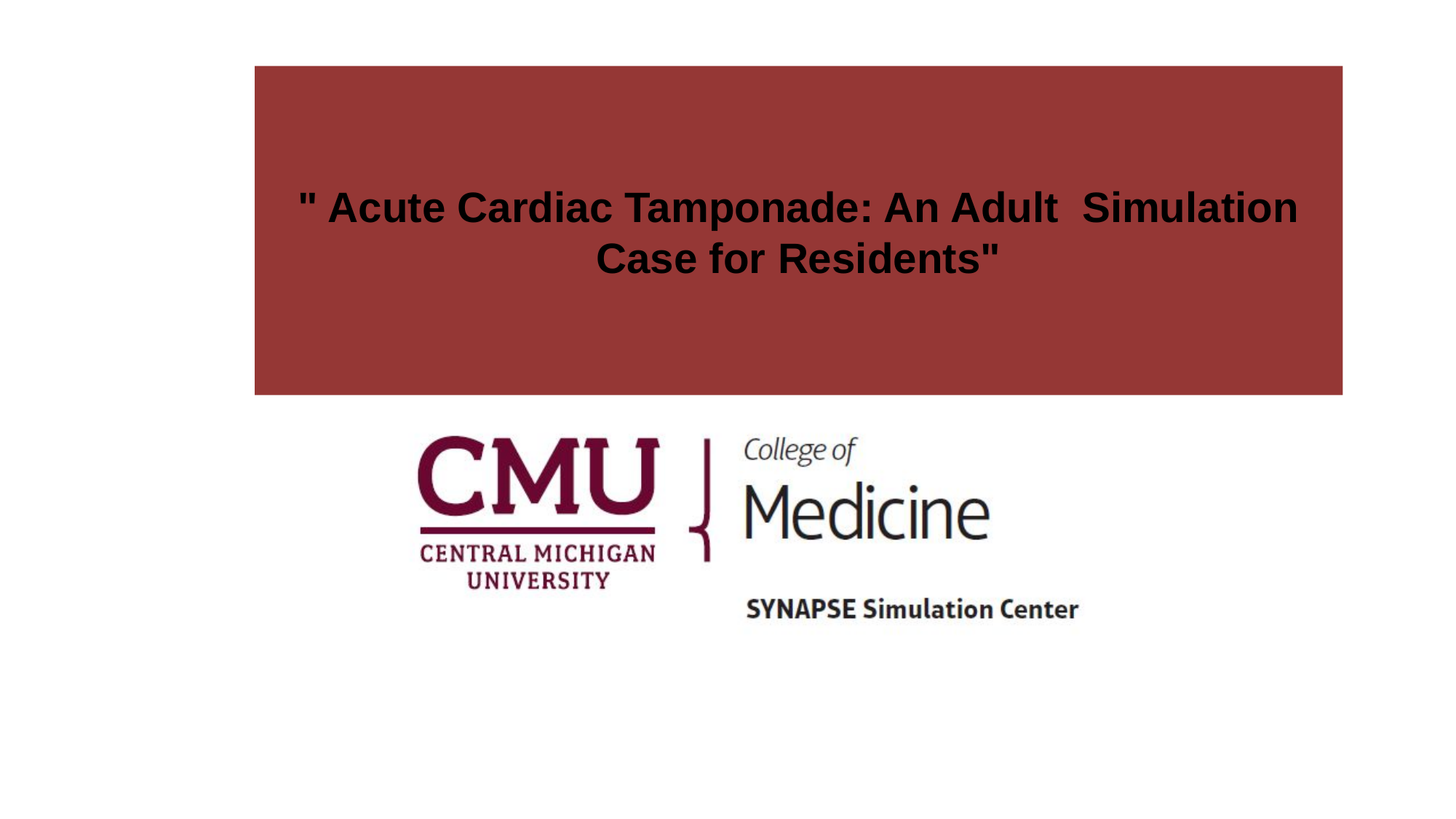

# " Acute Cardiac Tamponade: An Adult Simulation Case for Residents"

## Slide 2
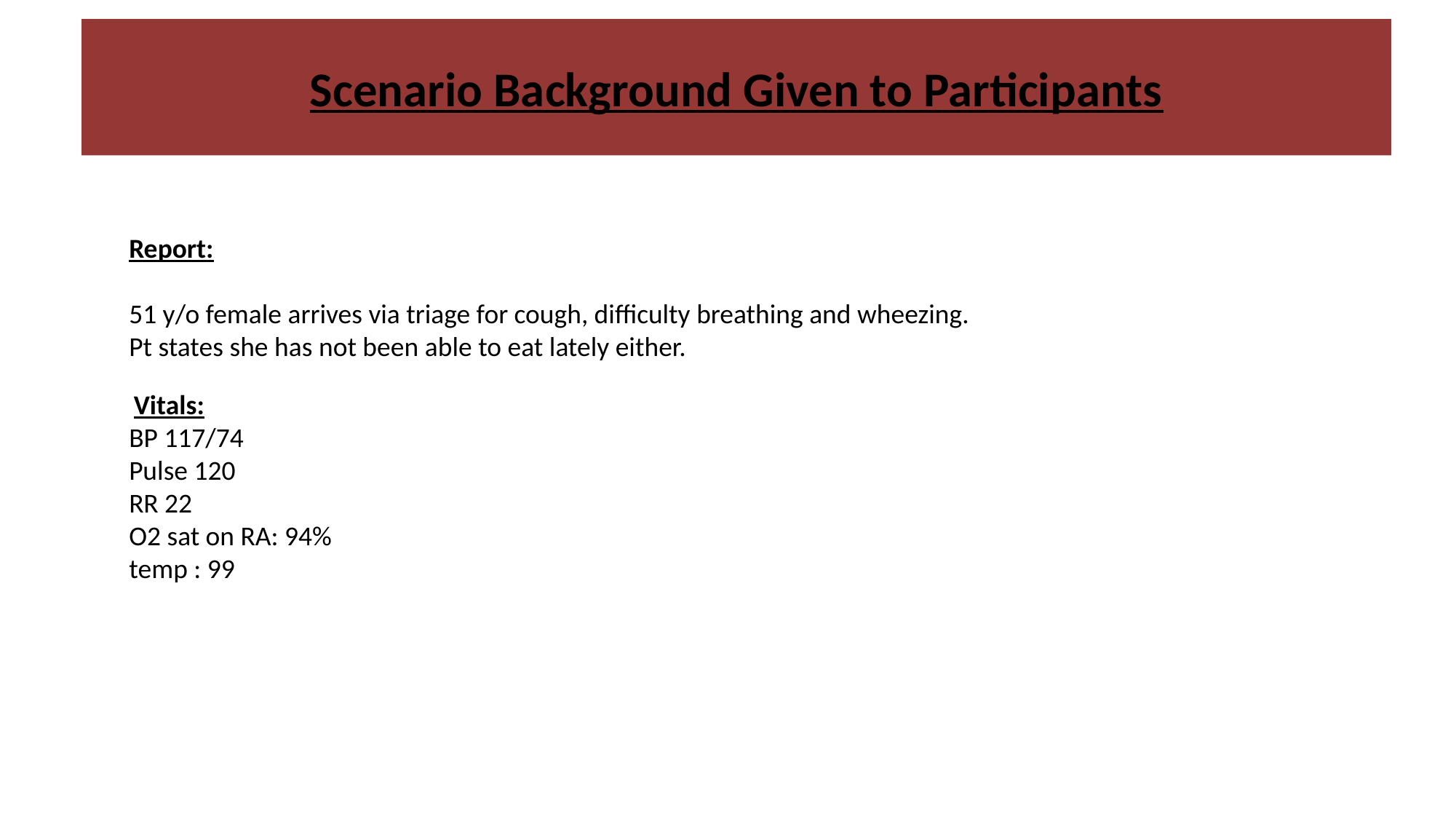

Scenario Background Given to Participants
Report:
51 y/o female arrives via triage for cough, difficulty breathing and wheezing. Pt states she has not been able to eat lately either.
 Vitals:
BP 117/74
Pulse 120
RR 22
O2 sat on RA: 94%
temp : 99

## Slide 3
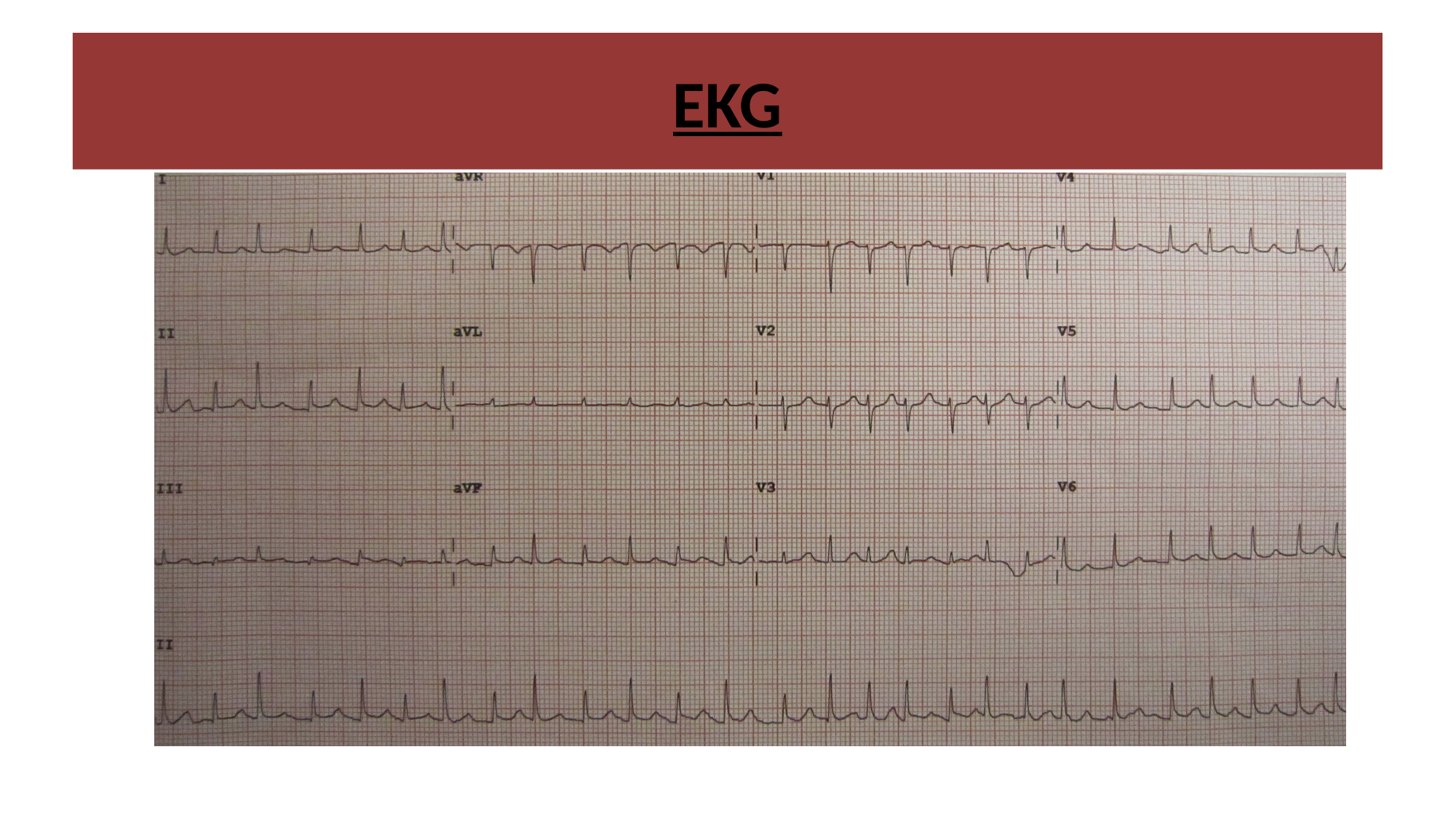

# EKG

## Slide 4
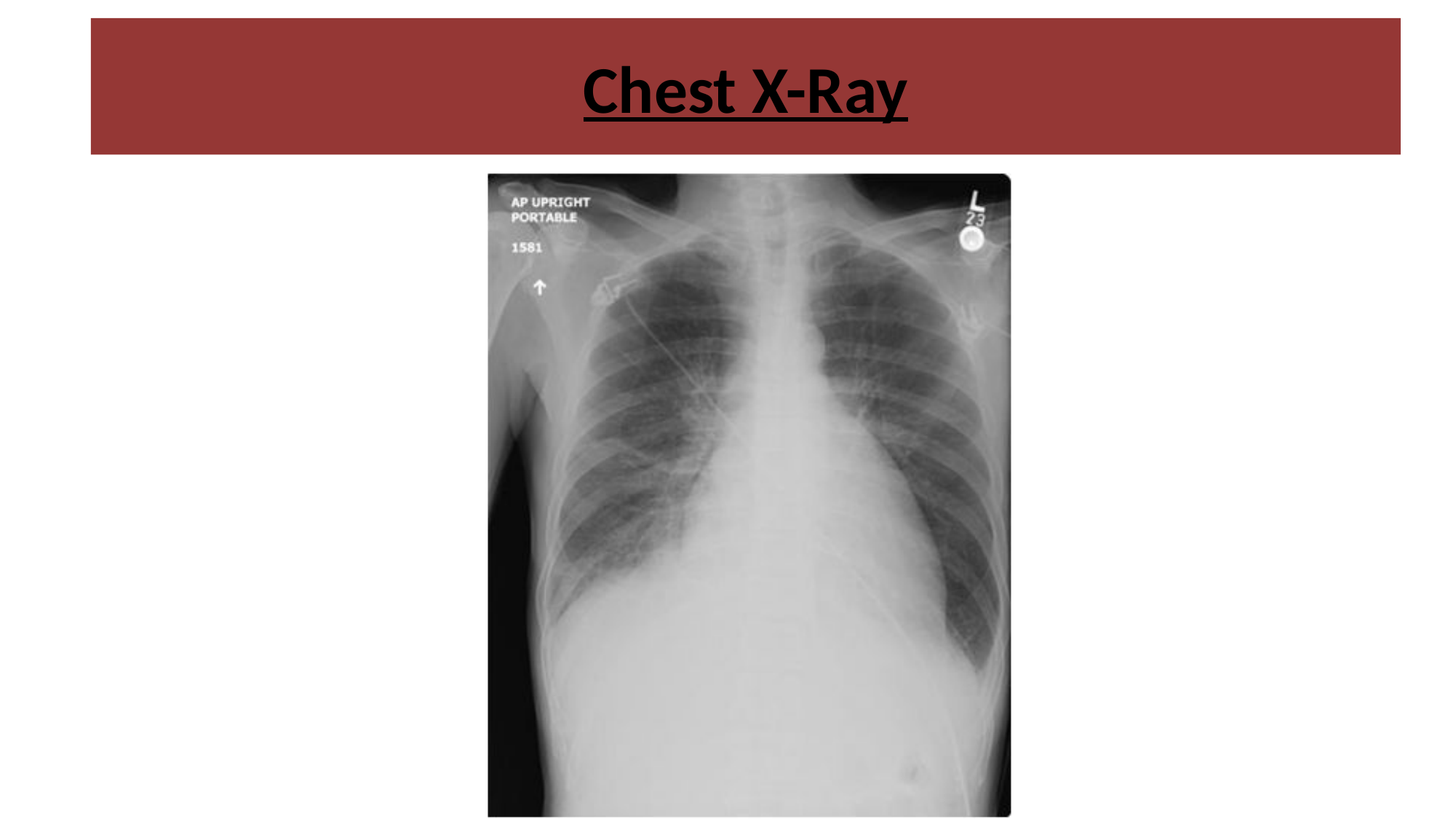

Chest X-Ray

## Slide 5
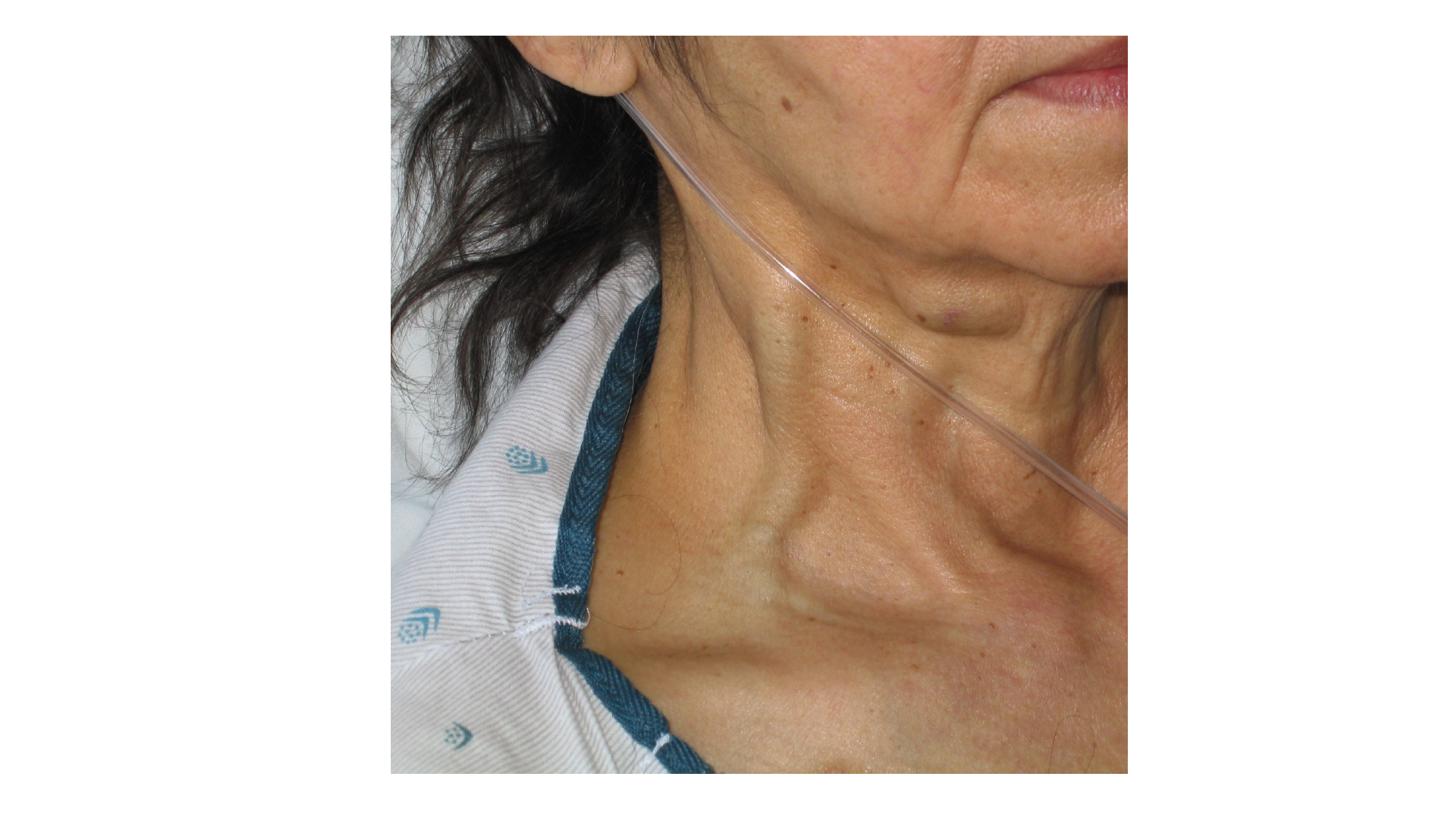

## Slide 6
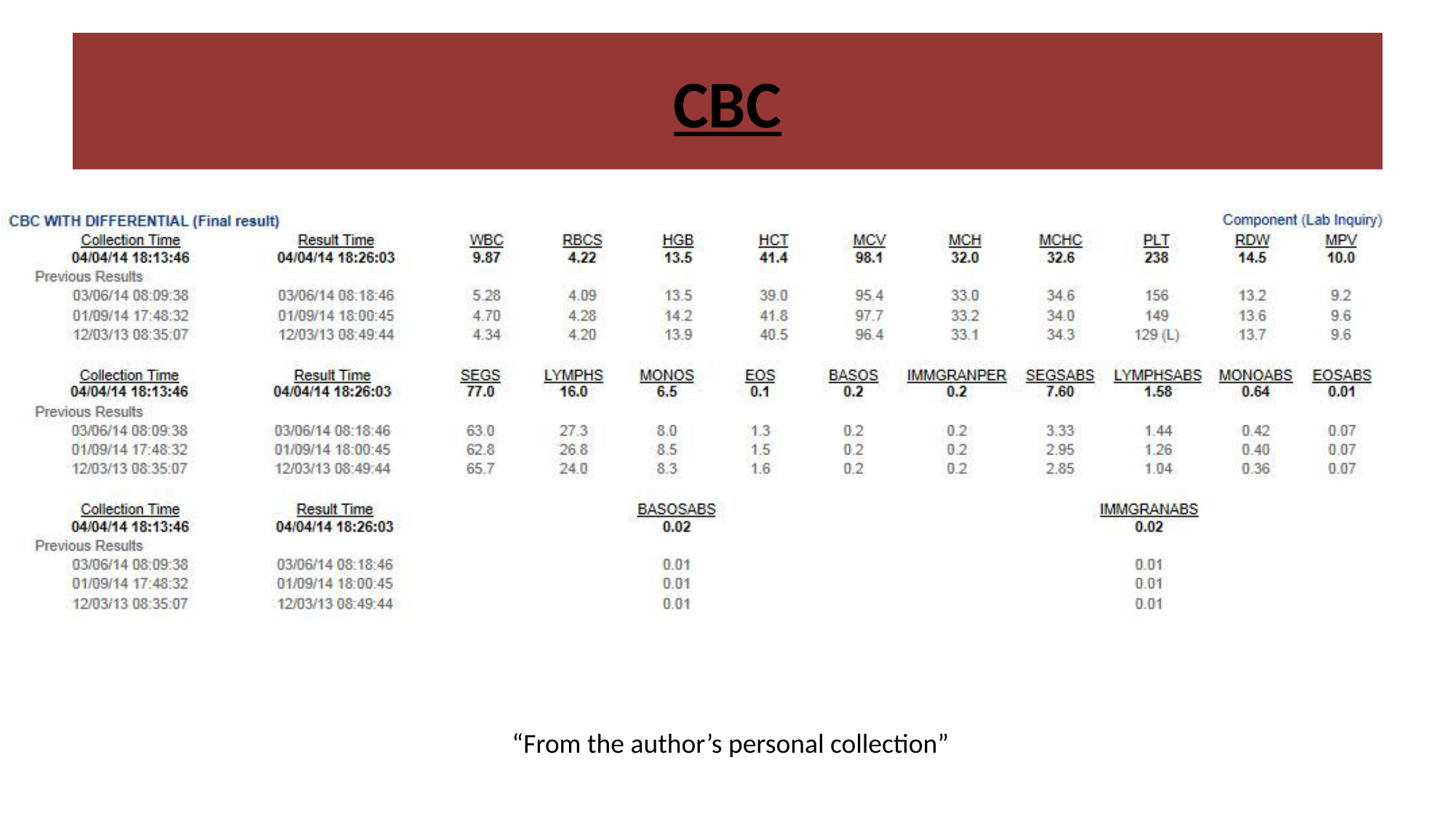

CBC
# CBC
 “From the author’s personal collection”

## Slide 7
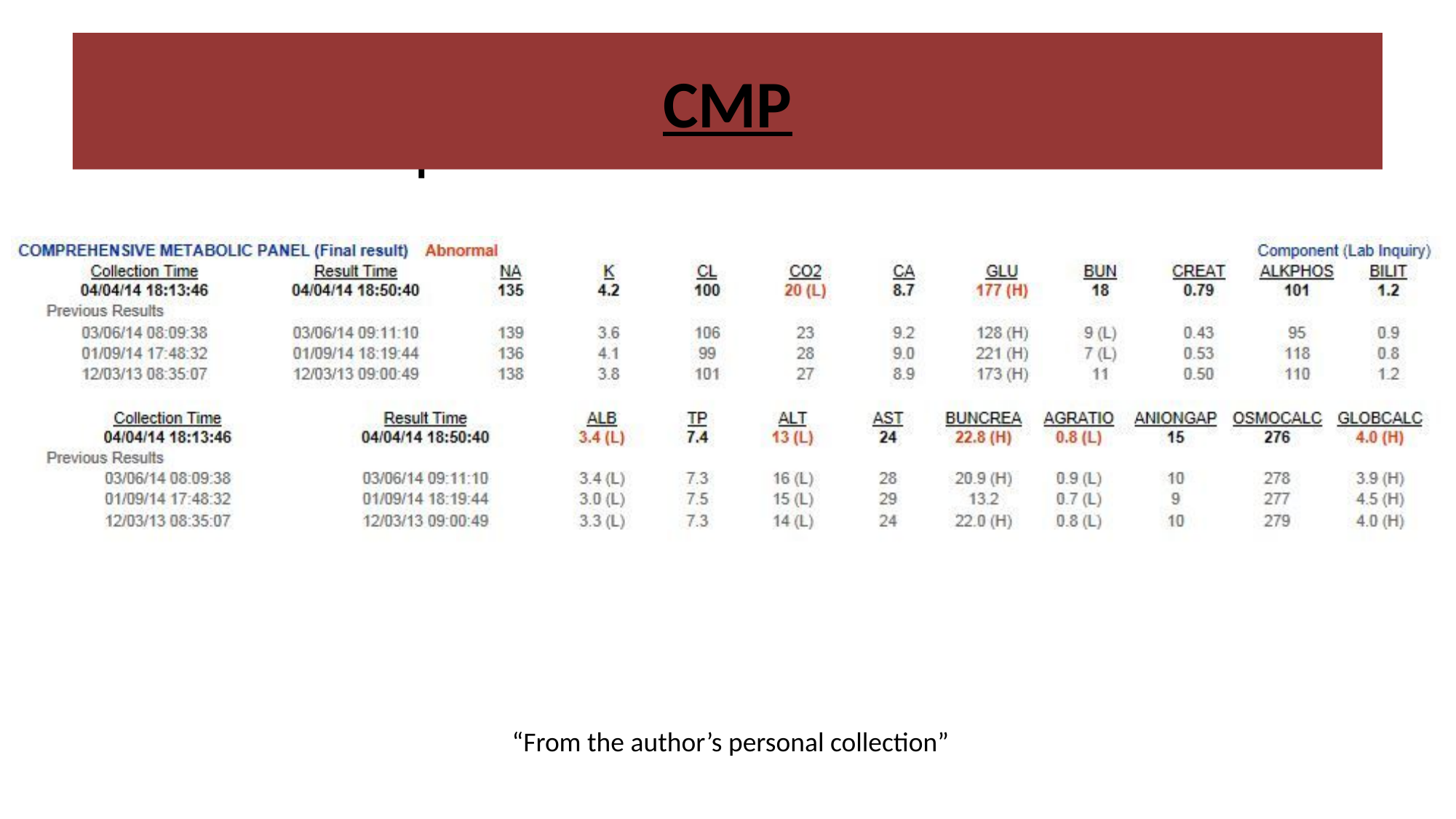

CMP
# Comprehensive Metabolic Panel
 “From the author’s personal collection”

## Slide 8
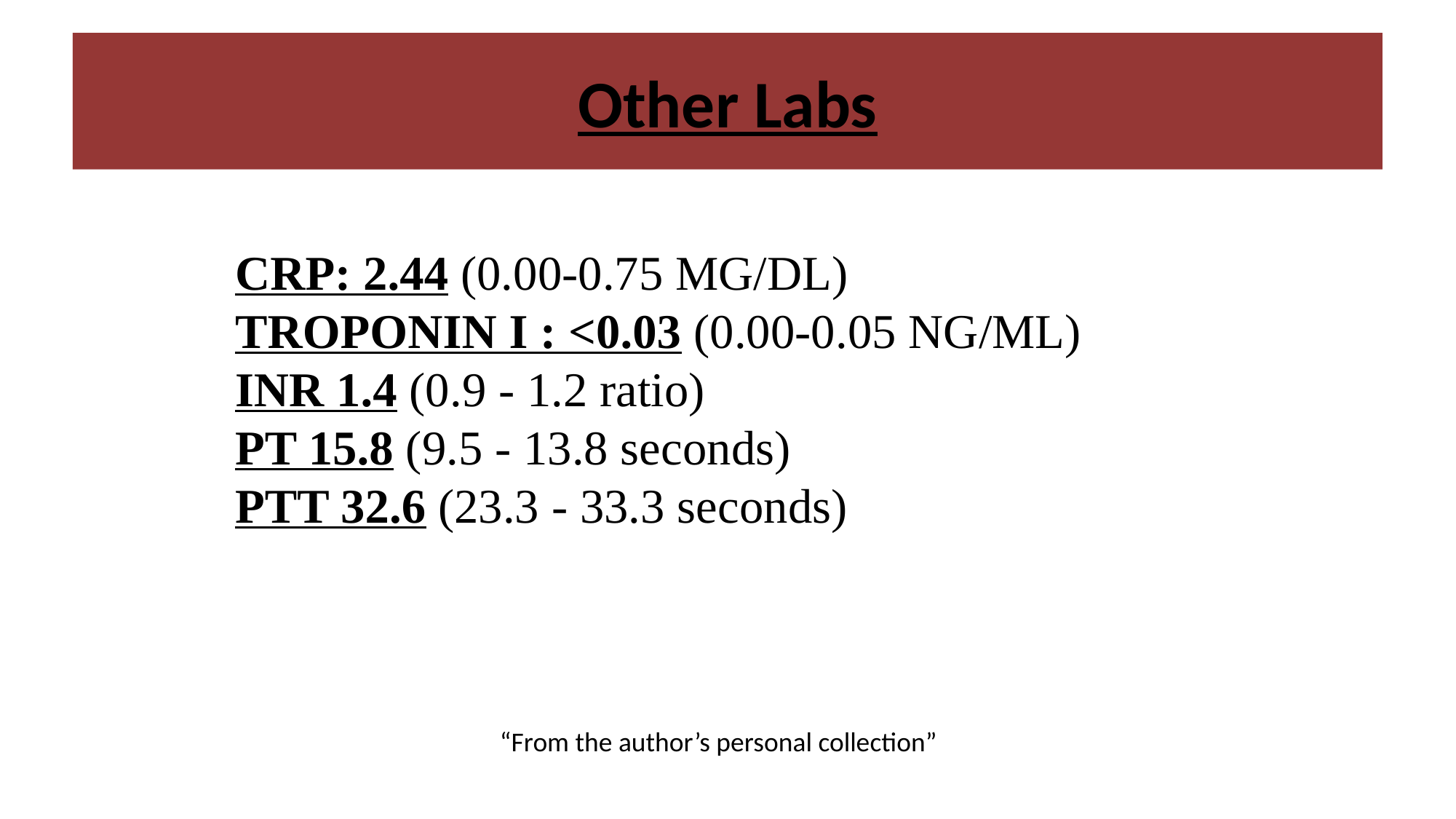

# Other Labs
CRP: 2.44 (0.00-0.75 MG/DL)
TROPONIN I : <0.03 (0.00-0.05 NG/ML)
INR 1.4 (0.9 - 1.2 ratio)
PT 15.8 (9.5 - 13.8 seconds)
PTT 32.6 (23.3 - 33.3 seconds)
 “From the author’s personal collection”

## Slide 9
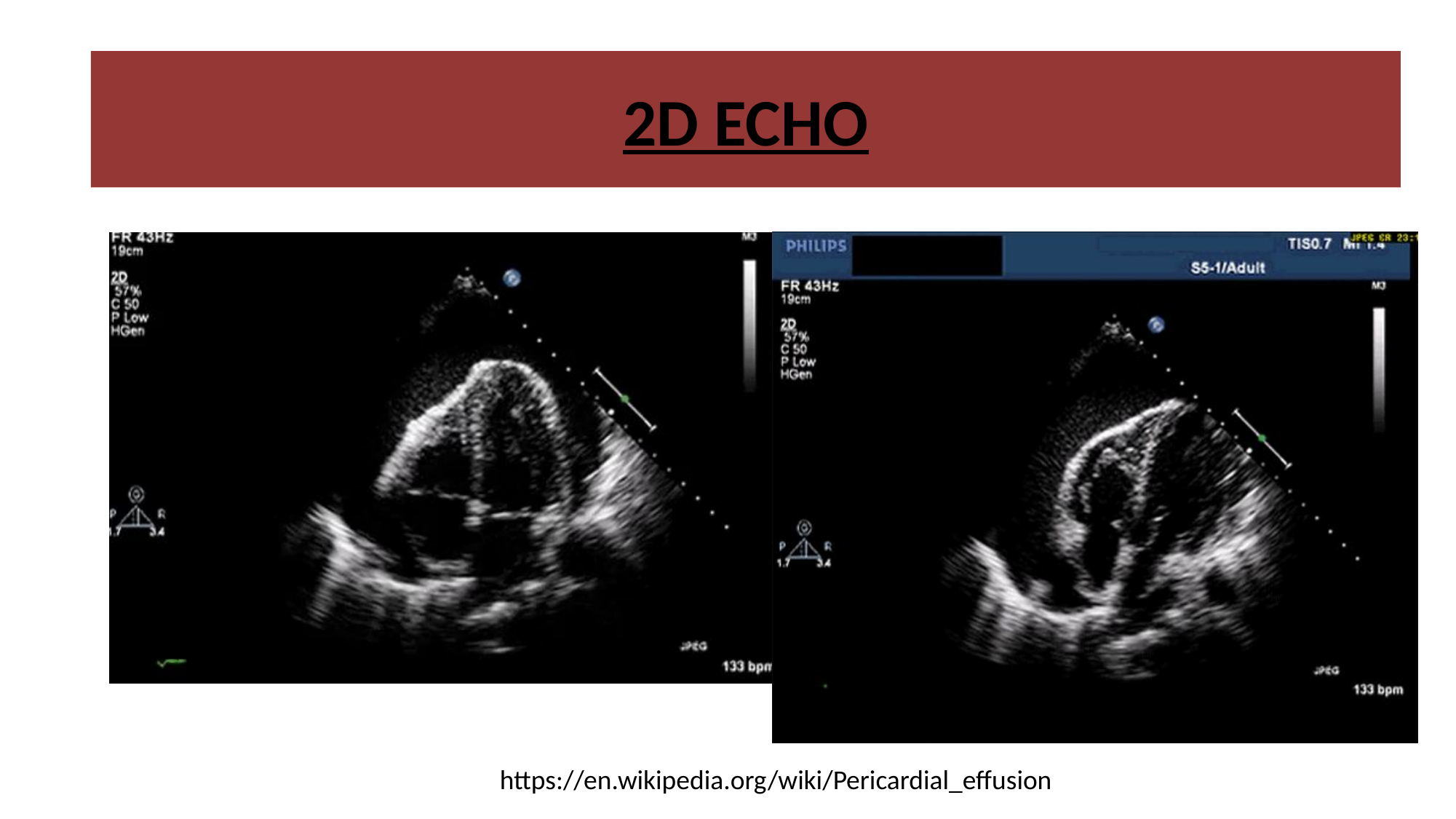

# Bedside 2D Echo
2D ECHO
https://en.wikipedia.org/wiki/Pericardial_effusion

## Slide 10
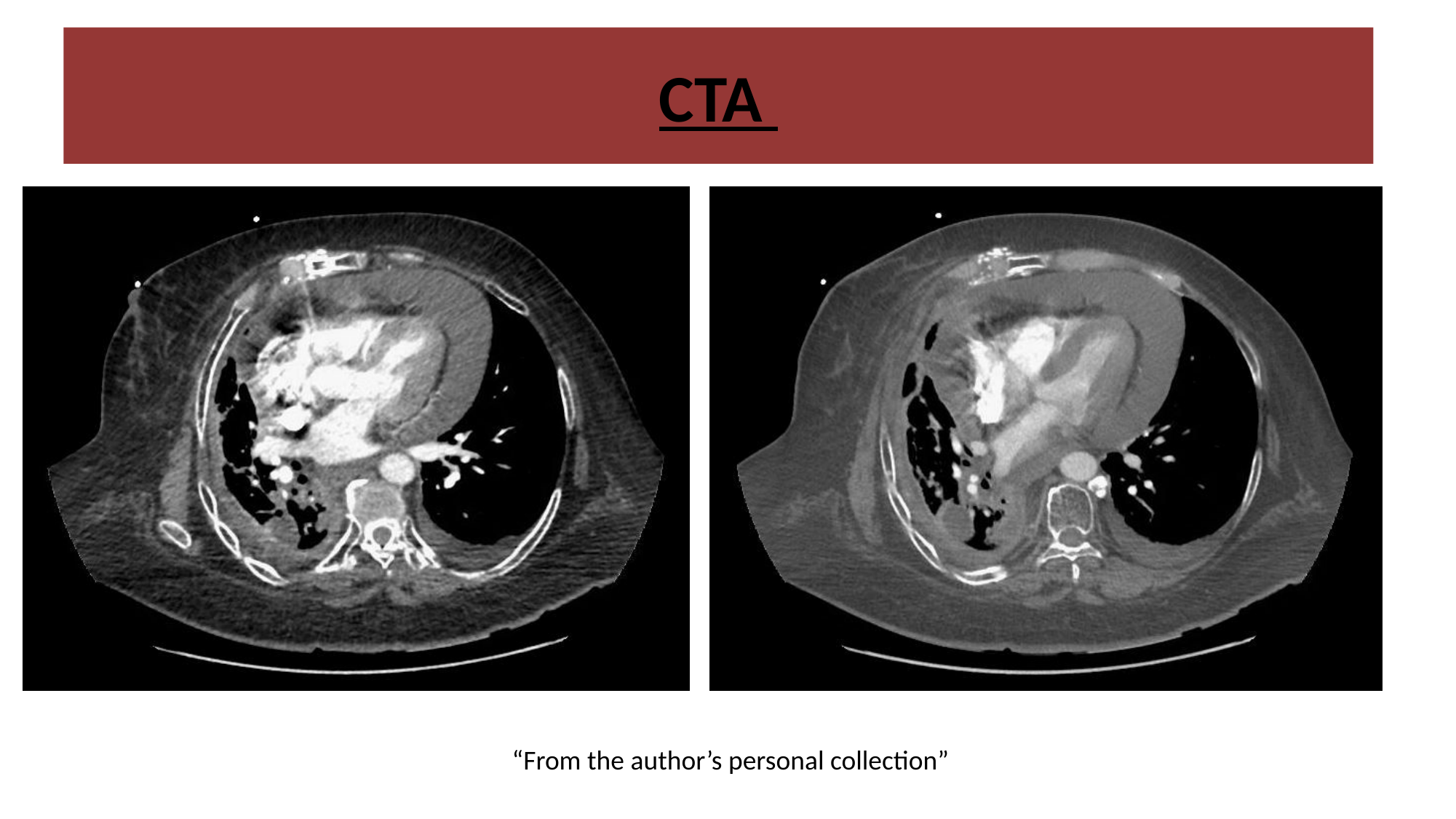

CTA
# CTA
 “From the author’s personal collection”

## Slide 11
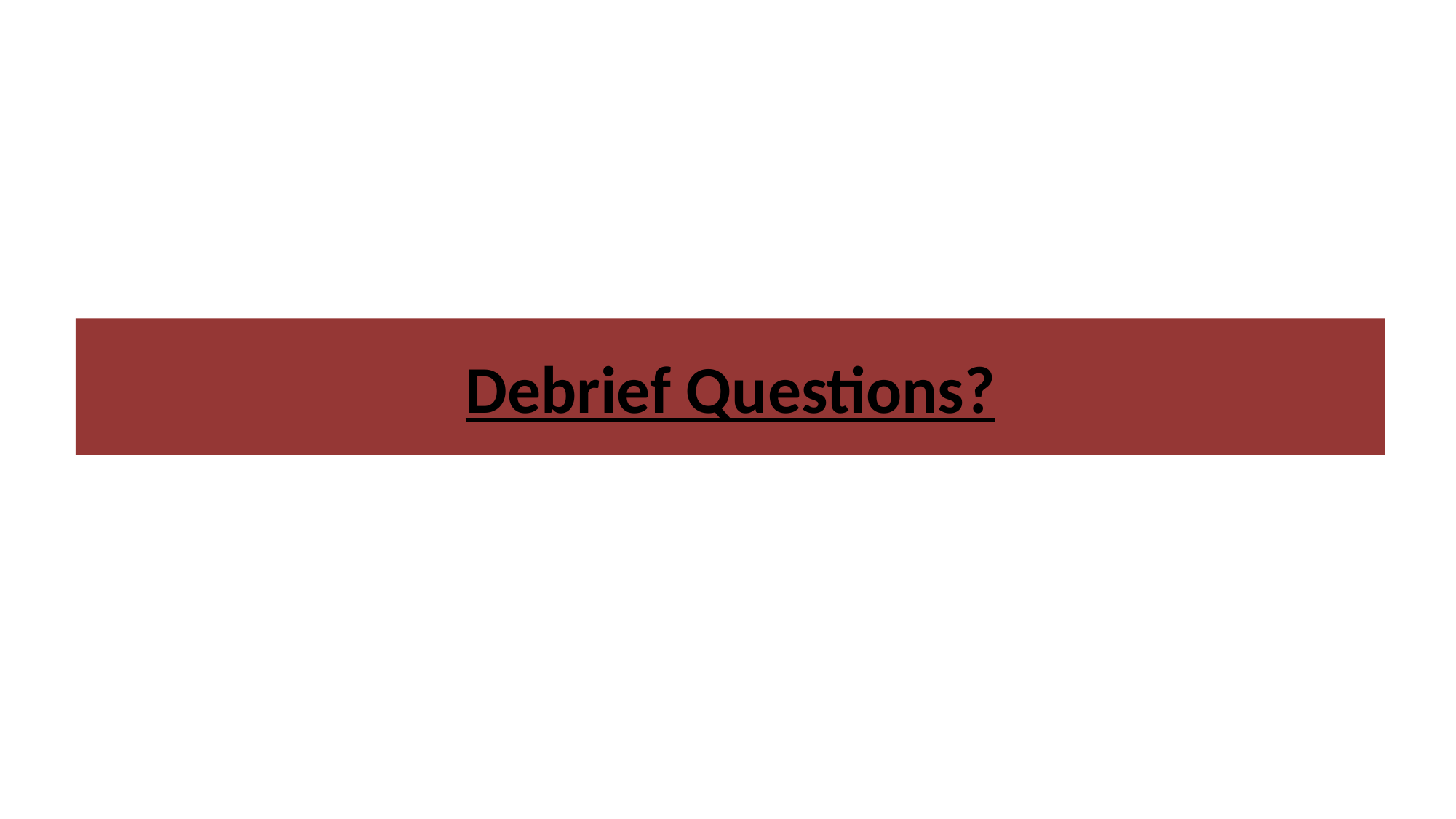

# Debrief Questions?

## Slide 12
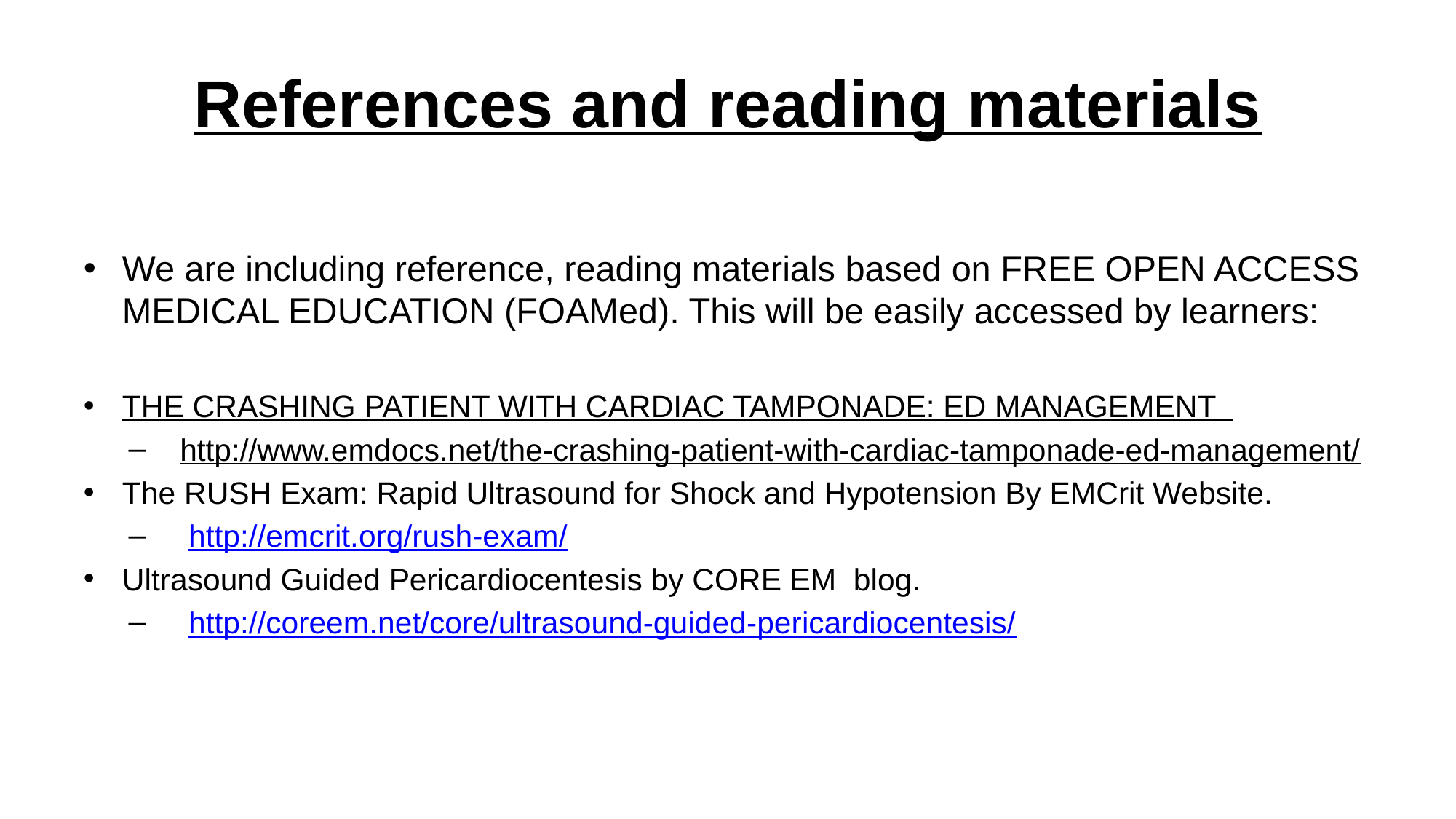

# References and reading materials
We are including reference, reading materials based on FREE OPEN ACCESS MEDICAL EDUCATION (FOAMed). This will be easily accessed by learners:
THE CRASHING PATIENT WITH CARDIAC TAMPONADE: ED MANAGEMENT
http://www.emdocs.net/the-crashing-patient-with-cardiac-tamponade-ed-management/
The RUSH Exam: Rapid Ultrasound for Shock and Hypotension By EMCrit Website.
 http://emcrit.org/rush-exam/
Ultrasound Guided Pericardiocentesis by CORE EM blog.
 http://coreem.net/core/ultrasound-guided-pericardiocentesis/
