# Supplementary material for: Acute Cardiac Tamponade: An Adult Simulation Case for Residents
Source: MedEdPORTAL. 2016 Sep 23;12:10466. doi: 10.15766/mep_2374-8265.10466 (PMC6464418; doi:10.15766/mep_2374-8265.10466)
Supplement: Supplementary file 1 — A. Simulation Case.docx B. PowerPoint Presentation.pptx C. Critical Actions Checklist.docx D. Assessment of a Low-Cost Ultrasound Pericardiocentesis Model.pdf [file mep-12-10466-s001.zip › C. Critical Actions Checklist.docx]

*Appendix C*: **Critical actions checklist**

1. **Evaluate** airway, breathing and circulation.
2. **Apply** cardiac monitor, pulse oximeter to patient.
3. **Establish** peripheral access.
4. **Apply** supplemental oxygen.
5. **Obtain** Past Medical History.
6. **Obtain** complete physical examination.
7. **Analyze** the significance of pertinent physical examination findings including**:** tachycardia, hypotension, distant heart sounds, and JVD**.**
8. Order stat portable chest x-ray.
9. **Recognize** significant cardiomegaly.
10. **Order EKG.**
11. **Recognize and discuss** electrical alternans.
12. **Perform** bedside ECHO.
13. **Interpret** ECHO, recognize pericardial effusion.
14. **Perform** emergent pericardiocentesis to stabilize patient with tamponade physiology. Learner should consult specialist if unfamiliar with the procedure.
15. **Admit patient to ICU,** contact appropriate consultants, cardiology, and cardiothoracic surgery**.**
